# Co-development of a transitions in care bundle for patient transitions from the intensive care unit: a mixed-methods analysis of a stakeholder consensus meeting

**DOI:** 10.1186/s12913-021-07392-2

**Published:** 2022-01-02

**Authors:** Brianna K. Rosgen, Kara M. Plotnikoff, Karla D. Krewulak, Anmol Shahid, Laura Hernandez, Bonnie G. Sept, Jeanna Morrissey, Kristin Robertson, Nancy Fraser, Daniel J. Niven, Sharon E. Straus, Jeanna Parsons Leigh, Henry T. Stelfox, Kirsten M. Fiest

**Affiliations:** 1 Department of Critical Care Medicine, University of Calgary, 3260 Hospital Drive NW, Calgary, Alberta T2N 4Z6 Canada; 2 Department of Community Health Sciences, University of Calgary, 3280 Hospital Dr NW, Calgary, AB T2N 4Z6 Canada; 3 Critical Care Strategic Clinical Network, Alberta Health Services, 10030 – 107 Street NW, Edmonton, AB T5J 3E4 Canada; 4 Department of Medicine, Institute for Health Policy, Management and Evaluation, University of Toronto, 1 King’s College Cir, Toronto, ON M5S 1A8 Canada; 5 Department of Medicine, School of Health Administration, Dalhousie University, 1276 South Park Street, Halifax, NS B3H 2Y9 Canada; 6 O’Brien Institute for Public Health, 3280 Hospital Dr NW, Calgary, AB T2N 4Z6 Canada; 7 Department of Psychiatry, Cumming School of Medicine, University of Calgary, 2500 University Drive NW, Calgary, AB T2N 1N4 Canada

**Keywords:** Critical care, Patient discharge, ICU, Adult, Transitions in care

## Abstract

**Background:**

Intensive care unit (ICU) patients undergoing transitions in care are at increased risk of adverse events and gaps in medical care. We evaluated existing patient- and family-centered transitions in care tools and identified facilitators, barriers, and implementation considerations for the application of a transitions in care bundle in critically ill adults (i.e., a collection of evidence-based patient- and family-centred tools to improve outcomes during and after transitions from the intensive care unit [ICU] to hospital ward or community).

**Methods:**

We conducted a concurrent mixed methods (quan + QUAL) study**,** including stakeholders with experience in ICU transitions in care (i.e., patient/family partners, researchers, decision-makers, providers, and other knowledge-users). First, participants scored existing transitions in care tools using the modified Appraisal of Guidelines, Research and Evaluation (AGREE-II) framework. Transitions in care tools were discussed by stakeholders and either accepted, accepted with modifications, or rejected if consensus was achieved (≥70% agreement). We summarized quantitative results using frequencies and medians. Second, we conducted a qualitative analysis of participant discussions using grounded theory principles to elicit factors influencing AGREE-II scores, and to identify barriers, facilitators, and implementation considerations for the application of a transitions in care bundle.

**Results:**

Twenty-nine stakeholders attended. Of 18 transitions in care tools evaluated, seven (39%) tools were accepted with modifications, one (6%) tool was rejected, and consensus was not reached for ten (55%) tools. Qualitative analysis found that participants’ AGREE-II rankings were influenced by: 1) language (e.g., inclusive, balance of jargon and lay language); 2) if the tool was comprehensive (i.e., could stand alone); 3) if the tool could be individualized for each patient; 4) impact to clinical workflow; and 5) how the tool was presented (e.g., brochure, video). Participants discussed implementation considerations for a patient- and family-centered transitions in care bundle: 1) delivery (e.g., tool format and timing); 2) continuity (e.g., follow-up after ICU discharge); and 3) continuous evaluation and improvement (e.g., frequency of tool use). Participants discussed existing facilitators (e.g., collaboration and co-design) and barriers (e.g., health system capacity) that would impact application of a transitions in care bundle.

**Conclusions:**

Findings will inform future research to develop a transitions in care bundle for transitions from the ICU, co-designed with patients, families, providers, researchers, decision-makers, and knowledge-users.

**Supplementary Information:**

The online version contains supplementary material available at 10.1186/s12913-021-07392-2.

## Background

Transitions in care are transfers of a patient to another healthcare setting, involving hand off to another team of healthcare providers [1]. For intensive care unit (ICU) patients, transitions in care may involve transfer to an inpatient ward, long term care facility, or home [2]. Research has demonstrated that patients undergoing transitions in care are at increased risk of adverse events [3–5], and often experience lapses in communication between healthcare providers and gaps in medical care [6–9]. ICU patients may be especially vulnerable to poor outcomes during or after transitions in care because of the severity and complexity of their illness [10].

Patients, families, clinicians, and organizations have different needs during transitions in care [11–14]. Research has identified that patients and families face challenges during transitions from ICU, including worry and uncertainty, perceived gaps in care, and unfulfilled needs for more information [15]. Similarly, challenges from the perspective of ICU and ward staff have been identified, including lack of collaboration between units, delays in information transfer, incomplete or inaccurate information, and failure to engage patients and families throughout the transfer [14]. Strategies to address challenges faced by stakeholders involved in transitions in care must be multimodal to address the information and psychosocial needs of patients and families, and the structural (e.g., discharge plan), and process (e.g., time of transition, communication between providers and care settings) needs of healthcare providers and organizations.

A previous systematic review identified 47 candidate tools (i.e., an instrument that collects or delivers information) to facilitate transitions in care from the ICU, of which 18 were patient- and family-centered [1]. These included tools that target different phases of the discharge process (e.g., readiness for discharge, plan for discharge, execution of discharge, and post-discharge follow-up). To the best of our knowledge, the peer reviewed literature does not include a single tool that addresses all needs of patients, families, healthcare providers, and other relevant stakeholders. Instead, these individual tools may be combined to form the basis of a transitions in care bundle, envisioned to be a collection of evidence-based, patient- and family-centered tools for use by clinicians, patients, and family members that aim to improve patient and health system outcomes throughout the transitions in care process. To evaluate these existing tools and contribute to the creation of a transitions in care bundle, we hosted a meeting with stakeholder groups relevant to ICU transitions in care. The overall meeting goals were to: 1) evaluate existing patient- and family-centered transitions in care tools from the previously conducted scoping review for inclusion in a transitions in care bundle [1]; 2) facilitate multi-stakeholder dialogue on the overall assessment and modifications required to make the tools applicable to a critically ill adult population; and 3) identify facilitators, barriers, and implementation considerations for a transitions in care bundle. The findings of this meeting will inform the development and implementation of a transitions in care bundle.

## Methods

### Ethical considerations and design

We conducted a concurrent mixed methods study (quan + QUAL), reported according to the Standards for Reporting Qualitative Research (SRQR) guidelines. A one-day meeting was attended by stakeholders involved in ICU patients’ transitions in care in Calgary, Alberta on September 5, 2019. This included 29 individuals (7 patient partners [including family members of former ICU patients]; 10 researchers [3 of whom had a secondary role as a healthcare provider]; 5 healthcare providers; 4 knowledge-users [e.g., those in roles that influence standard of care practices that may also have clinical roles]; 3 decision-makers [e.g., those in roles who can effectively implement new changes to healthcare]). Patient partners, researchers, and healthcare providers were purposefully recruited through the Critical Care Strategic Clinical Network and the Seniors Health Strategic Clinical Network, aiming for diversity across stakeholder groups. Representative decision makers and knowledge-users were nominated by the Canadian Critical Care Society. This meeting received ethical approval by the University of Calgary’s Conjoint Health Research Ethics Board (REB17–0027). Meeting participants provided informed written consent prior to participating.

### Meeting activities

The meeting included three components. First, a presentation was given summarizing existing transitions in care tools and how to employ the modified Appraisal of Guidelines, Research and Evaluation (AGREE-II) criteria to evaluate assigned tools (Additional file 1) [16]. Second, participants were divided into three groups (5–6 participants per group), with representatives from each key stakeholder group, and assigned 6 patient- and family-centered transitions in care tools identified by the previous scoping review (sent in advance of the meeting) [1]. Prior to the meeting, participants independently rated each tool using the modified AGREE-II criteria which uses a 7-point Likert scale from 1 (strongly disagree) to 7 (strongly agree) [16]. During the AGREE-II scoring review at the meeting, participants discussed whether or not they would recommend the tools for use (i.e., Yes/No/Yes, but with modifications). Research assistants collected and tabulated the scores. If consensus was not reached for a tool (i.e., < 70% of participants either did or did not recommend the tool), it was discussed by the group to understand why some felt it was or was not a good fit for transitions in care of critically ill adults. Tools that participants recommended with modifications were discussed to understand what changes the tool required. Third, meeting participants were divided into six groups (different groups from the tool scoring exercise, with 4–5 participants per group), with representatives from each key stakeholder group to discuss implementation considerations (e.g., format of the transitions in care bundle, additional materials), and facilitators and barriers to the application of a transitions in care bundle (Additional file 2). Participants recorded key points of the discussion on poster boards for all participants to see and comment further. The meeting was audio recorded and research assistants took notes during breakout sessions and deliberations to share ideas with the broader group.

### Data analysis

We summarized the modified AGREE-II rankings of the existing transitions in care tools using medians and interquartile range (IQR). Audio recordings of the meeting were transcribed verbatim, reviewed for accuracy, de-identified, and imported into NVivo-12 (QSR International, Melbourne, Australia) for data analysis. Data were analyzed using principles of grounded theory, including open and axial coding [17]. Three research assistants (BR, KP, LH) iteratively completed open coding, independently and in duplicate, meeting frequently to compare emerging themes, and ensure consistent application of codes [17]. Following the completion of open coding, reviewers (AS, BR, KP) met to develop a refined coding library, using principles of axial coding [17]. Two reviewers (AS, BR) applied the refined coding library to all transcripts, grouping coded quotes into the themes and subthemes.

## Results

Of the 29 stakeholders, 27 (93.1%) evaluated existing transitions in care tools. Thirteen of the 18 tools (72%) evaluated were intended for use in pediatric and neonatal populations. Tools included discharge summaries, worksheets, checklists, and educational tools, which were designed for use by staff (*n* = 14, 78%), or staff and patients and families (*n* = 4, 22%), with patients and families (*n* = 18, 100%) as the target receiver population. Seven (39%) tools were accepted with modifications, one (6%) tool was rejected, and consensus was not reached for ten (55%) tools (Additional file 1).

The qualitative analysis revealed that participants reported that their decisions for AGREE-II rankings of existing transitions in care tools were based on five main areas: 1) language used (e.g., inclusive, positive, balance of jargon and lay language); 2) if the tool was comprehensive to effectively facilitate transitions in care of critically ill adults (i.e., could be used alone); 3) if the tool could be individualized for a critically ill adult’s unique needs; 4) how the tool impacted workflow; and 5) how the tool was presented (e.g., brochure, video, cartoon). Several tools were deemed too specific and “*didn’t seem like [they were] going to be easily transferred into an adult ICU environment*” (−Healthcare Provider) (e.g., “Discharge Planning for AIDS Patients” [18] or “Safety Checklist for Discharge Planning” [19]) or “*felt like a framework*” (−Healthcare Provider) for creating a tool instead of a tool that was ready to use (e.g., “Information Booklet,” [20] “FICare,” [21] “Project CONNECT” [22]). For example, the “FICare” tool outlined general strategies for family-integrated care teaching, such as “[...] We must be positive and have an empathetic concern for our families have respect for us as healthcare providers and that we have respect for our families. We must be positive and have an empathetic concern for the individuality of the learner.”

Participants ranked tools low (i.e., < 70% in AGREE-II ratings) when they “*did not see [the tool] as being patient- or family-oriented*” (−Patient Partner) (e.g., “Back Transport” [23]) or if they perceived that patients and families were not included in its development (e.g., “FICare,” [21] “Project CONNECT” [22]). Most participants found the “Transfer Preparation Letter” [24] to include directed language about the patient instead of “*making it actively inclusive*” (−Patient Partner) of the patient and family. Participants ranked tools higher when patients and families were included in the transition process such as the “PBP for NICU Discharge Planning” [25, 26], which included patients and families in the tracking of the patient’s condition. Tools were ranked more favourably when positive language was used such as the “Discharge from the ICU to Ward Brochure,” [27] that justified patients moving to the ward “because they have improved – it is a positive step forward in your child’s recovery” , which participants felt could alleviate anxiety during this transition. Participants agreed that most of the transitions in care tools discussed could not stand alone when critically ill patients transitioned out of the ICU and instead could have “*value as part of a [...] package*” (−Knowledge-User). For example, several tools described the transition from the ICU to the ward (e.g., “Discharge from the ICU to Ward Brochure,” [27] “NICU Discharge DVD,” [28, 29] “Structured Transfer Brochure,” [30] “Transfer Preparation Letter” [24]), but did not provide information following the transition. Participants felt that a transitions in care bundle should include information that was addressed in other tools, such as components of a “continuing care plan” (“Project CONNECT” [22]), physiological abilities (e.g., “cardiovascular status”, “sleep patterns”, “gait/mobility”) (“Discharge Planning Questionnaire” [31, 32], “Pediatric Acute Burn Discharge Planning” [33]), “nutrition”,” medications”,” treatments” (e.g., “Nursing Case Management” [34]), and “prognosis or residual disability at discharge” (e.g., “Discharge Summary” [35]). Participants felt that any tool included in a transitions in care bundle must be adaptable to each patient’s unique care needs, which would not be possible with video-based tools (e.g., “NICU Discharge DVD” [28, 29]), but would be possible with tools that include general categories such as “Breathing,” “Feeding,” and “Growing” included in the “Discharge Planning Train” [36] or “Physiological, Nutritional, and Medication Evaluations” included in the “Pediatric Acute Burn Discharge Planning Index” [33].

Patient partners generally rated tools that included a visual component higher compared to tools without (e.g., “Nursing Care Management” [34] [cartoon] or “Discharge Planning Train” [36] [interactive visual indicator modelled as a train]). Though healthcare providers and decision-makers liked these tools because they identified patient milestones required for transfer, many worried about how it may “*concern the family*” (−Researcher) if a patient did not reach a milestone before transfer or if a patient’s condition regressed (e.g., milestone moved green [“ready for transfer”] to red [“requires care”]). Healthcare providers ranked tools low if they were lengthy, because “*completing [the tool] would be a huge amount of time and time away direct patient care and family care*” (−Knowledge-User) (e.g., “75-item NDAT” [37], “UCCDIP” [38], “Project CONNECT” [22]), but were more accepting of tools “*divided up into [categories]*” (−Decision-Maker) that could be completed by the multidisciplinary team or integrated with the electronic medical record (EMR) (e.g., “Pediatric Acute Burn Discharge Planning Index” [33]). For example, the “Pediatric Acute Burn Discharge Planning Index” [33] includes multiple categories that could be populated through existing information in the EMR, including “respiratory status”, “urological status”, “presence of infection”.

Twenty-four stakeholders (24/29, 82.8%), were divided into five groups that discussed implementation considerations, facilitators, and barriers to the application of a transitions in care bundle. Participants discussed delivery of the transitions in care bundle, continuity of care from the ICU to other settings, and continuous evaluation and improvement (Additional file 2). These aspects were also reflected in the conversations from the AGREE-II scoring review sessions. Healthcare providers, knowledge-users, and decision-makers noted that the transitions in care bundle should “*complement and be consistent with [the EMR]*” (−Knowledge User). Patient partners, healthcare providers, and decision-makers also shared that an electronic version (e.g., website or app) may be helpful, where patients and families can refer to information and include graphics or embedded videos, so that “*you know what happened to you*” (−Healthcare Provider). All participants discussed that an electronic-only version of the transitions in care bundle may not be accessible by all patients and families (e.g., if they do not have their own devices) and that the bundle should be available in multiple formats. Several participants suggested the transitions in care bundle be generated *from* the EMR as a means to integrate current infrastructure and workflow.

Identified facilitators are outlined in Additional file 2. All participants agreed that the transitions in care bundle should use plain language that is comprehensible to all users, including translation into other languages. Several healthcare providers suggested that medical jargon be included alongside plain language to familiarize patients and families with jargon potentially used in future conversations. Patient partners shared that it would be important for a member of the ICU care team to walk patients and families through the transitions in care bundle. “*Preferably, this ICU team member would have a close relationship with the family and present throughout their stay*” (−Patient Partner) (e.g., bedside nurse, social worker, or physician). Healthcare providers also shared that it’s important to have face-to-face interaction, to “*know how much [the patient and family] understand*” (−Patient Partner) and complement the textual information received.

Additionally, continuity of the transitions in care bundle past ICU discharge to the ward or community must be considered. Methods to achieve continuity discussed by participants included: follow-up from the ICU care team, family engagement, and community support systems (e.g., peer support groups). Following implementation of the transitions in care bundle, participants recommended continuous evaluation of the tools to achieve optimal performance and effectiveness. Participants added that this would be another benefit of an electronic transitions in care bundle, because the number of people (i.e., healthcare providers and patients or families) using it could be quantified by “*keeping track of audit feedback*” (−Researcher).

Participants suggested that the transitions in care bundle should be adaptable to varying geographic locations, with different levels of budget allocation and personnel resources (e.g., large urban tertiary hospitals versus small rural community hospitals). Additionally, the transitions in care bundle must be adaptable across patient conditions observed in the ICU (e.g., long versus short stays, complex versus less complex medical needs), given that “*patients in the ICU are very complex, very different*” (—Researcher). Participants agreed that the transitions in care bundle should facilitate collaboration between the patients, their families, and the ICU care team. This may be accomplished by including an input field dedicated to patient and family questions and comments. Additionally, participants highlighted the value of co-design between healthcare providers, researchers, and patients and families to the transitions in care bundle success, “*materials co-designed with patients who have that lived experience or family members [...] are going to be very relevant and probably more user friendly in many ways*” (−Decision-Maker).

Participants identified trust and transparency of information between patients, their families, and the ICU care team as a facilitator for transition in care bundle use. Similarly, healthcare providers and researchers identified transparency between different members of the healthcare team as a facilitator. In particular, healthcare providers identified that having the same information “*available to each member of the [multidisciplinary] team*” (−Healthcare Provider) could reduce communication lapses between care providers and consequent medical errors during a transition in care. Similarly, participants recommended that the “*information [in the bundle] remains consistent to what is being verbally said [by healthcare providers]”* (−Patient Partner) and information delivered in existing care pathways (i.e., existing discharge procedures and software systems).

Dedicated champions (i.e., internal leaders of change) for implementation of the transitions in care bundle could also facilitate successful implementation. One participant identified that “*there are champions who are trying to transform the system*” (−Healthcare Provider), who are reasons for success in existing areas of research in critical care. Champions may be members of the healthcare team or volunteers. Similarly, participants identified buy-in from healthcare providers as a facilitator to transitions in care bundle use.

Identified barriers are outlined in Additional file 2. Patient and family mental and emotional capacity was identified as a possible barrier to transitions in care bundle use. A potential way to overcome this is by employing the appropriate mode and timing of delivery. This includes having “*written [reference materials] [patients and families] could go back to later*” (−Patient Partner) and adequate follow-up by the healthcare team when capacity is regained.

Another patient- and family-oriented barrier was varying levels of family presence. While many ICU patients would benefit from tools that engage families at the bedside, these tools may have less benefit when there is “*no family [present at the bedside], which unfortunately is the case for some of the [ICU] patients*” (−Patient Partner). Healthcare system capacity was identified as a significant barrier due to existing constraints on healthcare providers’ workload, cost, and resources in the healthcare system. This coupled with health system culture (i.e., shared beliefs ingrained into healthcare systems) may also be a barrier to implementing changes in practice.

## Discussion

**Fig. 1:**
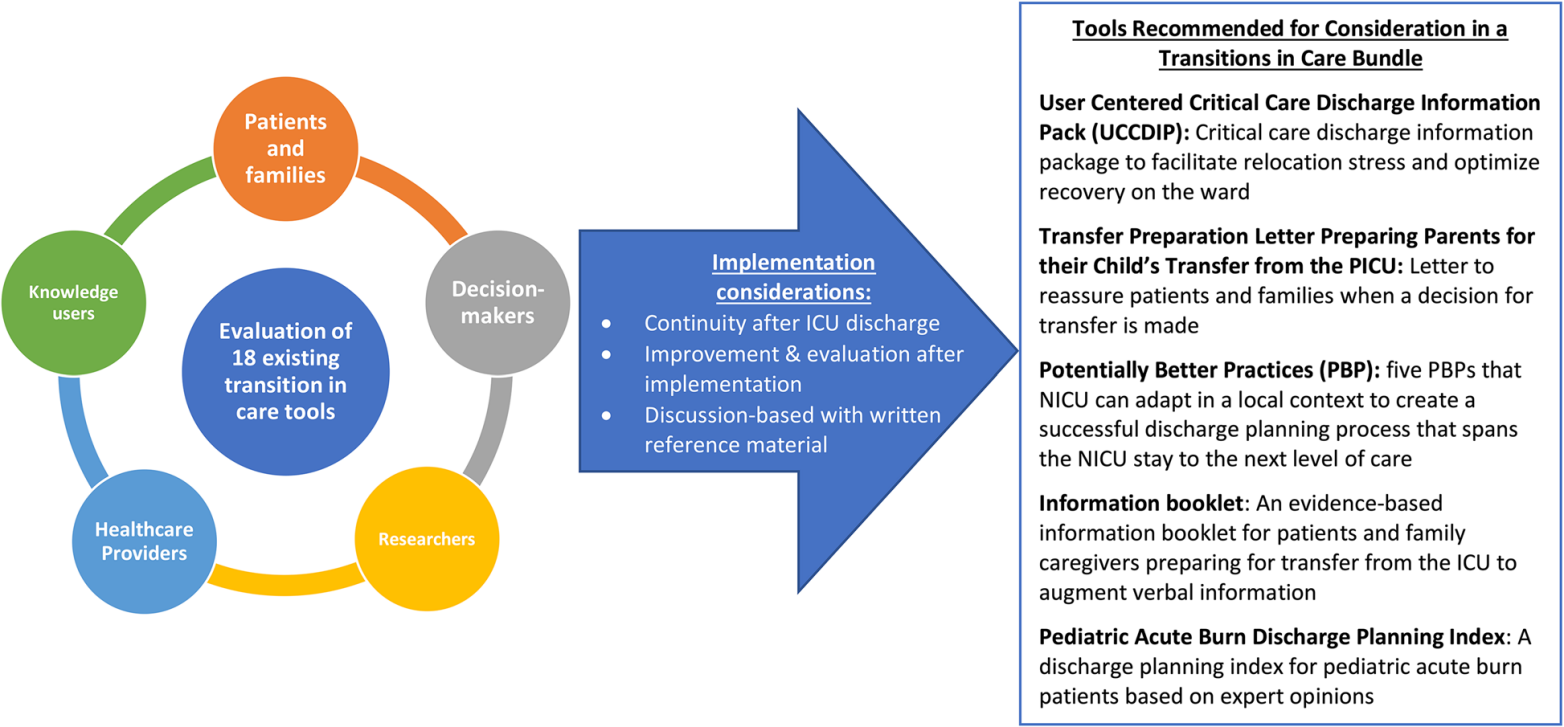
Roadmap of stakeholder groups engaged, implementation considerations, and resulting tools recommended for consideration in the creation of a new transitions in care bundle

This meeting united key stakeholders involved in transitions of care for critically ill adults, which is an important step toward co-designing a bundle to improve transitions in care from the ICU. During the review of existing patient- and family-centered transitions in care tools, consensus was achieved for less than half of the tools, with most categorized as acceptable but requiring modification before use in adult ICUs. Qualitative analysis revealed that participants scored tools based on the language used (e.g., balancing simple descriptions and medical jargon), the manner they were presented (e.g., brochure, video), and how they fit into current workflow. Participants identified several factors related to implementation of a transitions in care bundle and discussed barriers and facilitators during semi-structured focus groups. The key findings and recommendations from this study have been summarized in Fig. 1.

Though consensus was not achieved for most tools, the scoring exercise and discussion of existing transitions in care tools yielded rich perspectives from a multidisciplinary group of stakeholders. This is not unexpected, as most tools were intended for use in pediatric and neonatal populations. A qualitative study by *op ‘t Hoog* and colleagues identified that family caregivers of ICU patients desired acknowledgement from the healthcare team as a caregiver for the patient, briefing on expectations about transitions from ICU, and continuity of care for their critically ill loved one following discharge from ICU [39]. Stakeholders in our study similarly valued tools with an educational component for patients and families to understand expectations when leaving the ICU, and tools with an aspect of continuity following ICU discharge (e.g., follow-up after ICU discharge from the healthcare team or community supports).

Delivery format recommendations from our stakeholder meeting align with those from an existing study of cardiac patients discharged from hospital to home [40]. *Cawthon* and colleagues reported that 96% of participants found direct discussion with a pharmacist before hospital discharge helpful in managing medications [40]. Additionally, 92% found telephone follow-up from the care team helpful in managing medications after hospital discharge [40]. Our findings suggest that critically ill patients would also value direct discussion and follow-up during and after ICU transitions. Strategies for maximizing the quality of verbal patient and family communication have been proposed, which may be relevant for developing a high-quality patient- and family-oriented transitions in care bundle [41–43]. One example is patient-oriented discharge summaries, or PODS, which were implemented across Canada by The Canadian Foundation for Healthcare Improvement [44]. The discharge summaries were prepared by ICU staff, usually a nurse practitioner or physician, for patients being discharged directly home from the ICU. The PODS were reviewed with the patient, family, and by a member of the healthcare team before discharge, and included information about the patient’s stay and what should be done for follow-up after discharge [45].

### Future directions

The findings of the current study will be used to inform our team’s ongoing research, as well as future research in the broader field of ICU transitions in care. Our team will form a multidisciplinary working group (including patient and family partners, researchers, decision-makers, knowledge-users, and healthcare providers) to continue assessment of new transitions in care tools published since the stakeholder meeting. This will facilitate the creation, reiteration, and revision of the transitions in care bundle. We will identify and attempt to close gaps in existing patient- and family-oriented tools, centered around three engagement principles: inform, activate, and collaborate [46]. These principles will be used to ensure patients and family members receive information about their ICU stay, provide tools for patients and families to participate in care, and foster collaborative relationships between patients, family members, and members of the healthcare team, respectively [46]. Future research on ICU transitions in care is needed to modify existing transitions in care tools and develop new tools to incorporate themes identified in our study (Fig. 1), with emphasis on collaboration and co-design between healthcare providers, researchers, and patients and families. Further, prospective research is needed to evaluate the impact of such tools on patient outcomes (e.g., quality of life, satisfaction with care, functional capacity) and health system outcomes (e.g., readmission, length of stay). Through implementation of the findings from this study in our team’s ongoing work and in the broader field of ICU transitions in care, we hope to increase patient and family confidence, reassurance, and self-care while decreasing feelings of uncertainty and anxiety, ultimately empowering families to make decisions together with their loved ones and their care team.

### Limitations

This study had limitations to consider. Stakeholders were from a single health jurisdiction, which may limit generalizability to other healthcare jurisdictions. Although many target stakeholder groups were represented, there were few non-ICU healthcare providers (e.g., ward and primary care physicians) in attendance. These healthcare providers may have provided important insights as receivers of patients discharged from the ICU. Our team will address this by ensuring adequate representation of non-ICU healthcare providers in future work developing and evaluating the transitions in care bundle.

## Conclusions

This meeting brought together patient and family partners, healthcare providers, researchers, decision-makers, and knowledge-users to create a foundation for the development of a co-designed transitions in care bundle for improving transitions in care for patients discharged from the ICU. Through review of existing transitions in care tools, we identified that tools included in the bundle should have a visual element, have inclusive language that balances medical jargon and lay language, be adaptable for the heterogeneous ICU population, consider workflow, and be co-designed with relevant stakeholders impacted by transitions in care. Through multidisciplinary discussions, we identified implementation considerations, facilitators, and barriers to directly inform the implementation and evaluation of a transitions in care bundle in Canadian adult ICUs.

## Supplementary Information

**Additional file 1.**

**Additional file 2.**

## Data Availability

The data analyzed in the current study are not publicly available due ethical concerns but are available from the corresponding author on reasonable request.

## Abbreviations

ICU: Intensive care unit
SRQR: Standards for Reporting Qualitative Research
AGREE-II: Appraisal of Guidelines, Research and Evaluation
IQR: Interquartile range
EMR: Electronic medical record
PODS: Patient-oriented discharge summaries
