# Supplementary material for: Co-development of a transitions in care bundle for patient transitions from the intensive care unit: a mixed-methods analysis of a stakeholder consensus meeting
Source: BMC Health Serv Res. 2022 Jan 2;22:10. doi: 10.1186/s12913-021-07392-2 (PMC8722038; doi:10.1186/s12913-021-07392-2)
Supplement: Supplementary file 1 — Additional file 1. [file 12913_2021_7392_MOESM1_ESM.docx]

**Additional file 1.** Summary results of each tool evaluated by the Modified AGREE-II scale

|  |  |  |  | **AGREE-II Score** | | **Overall AGREE-II ranking** | | |  |
| --- | --- | --- | --- | --- | --- | --- | --- | --- | --- |
| **Tool Name** | **User** | **Target Population** | **Summary of tool** | **Median Total Score (IQR)** | **Median Overall Score (IQR)** | **Yes, would recommend n/N (%)** | **Yes, with modifications**  **n/N (%)** | **No, would not recommend**  **n/N (%)** | **Summary of discussion** |
| User Centered Critical Care Discharge Information Pack (UCCDIP) [37] | ICU staff | ICU patients and families | Critical care discharge information package to facilitate relocation stress and optimize recovery on the ward for patients and their family caregivers following transfer/discharge from ICU | 60 (56,63) | 6 (5,6) | 2/10 (20) | 8/10 (80) | 0/10 (0) | Participants liked that the UCCDIP can be personalized and included a lay patient discharge summary. However, they felt that the UCCDIP is targeted to providers, would be onerous to complete, and does not integrate with current systems. |
| Transfer Preparation Letter Preparing Parents for their Child’s Transfer from the PICU [23] | PICU staff | Patients and families | PICU nurse driven intervention to reduce stress in parents preparing for their child’s transfer to general, medical inpatient units | 44 (40,45) | 6 (5,6) | 1/9 (11.1) | 5/9 (55.6) | 3/9 (33.3) | Participants felt that this may be a nice letter to reassure patients and families when a decision for transfer is made (i.e., not provided to patients and families at the time of transfer). However, they disliked that this did not have inclusive language (i.e., including patients and families as part of the care team) and felt that this would not standalone as a TiC tool. |
| Family Integrated Care (FICare) model parent education program [20] | Patients and families, educational staff | Patients and families | Education program to support NICU parent’s participation in a pilot study of FICare | 36 (33,37) | 4 (2,5) | 1/8 (12.5) | 2/8 (25) | 5/8 (62.5) | Though participants conceptually liked the idea of teaching families to care for the patient when they leave the ICU, they perceive that this NICU tool is not applicable to a heterogeneous, critically ill adult population (i.e., not easily individualized). Moreover, they felt that patients and families were not involved in the creation and use of the tool. |
| Project CONNECT [21] | NICU staff | NICU patients and families | A multi-tiered caregiver engagement strategy aiming to improve and expand the implementation of coordinated, family-centered, community-based practices for families transitioning from NICU to home | 40 (37,43) | 5 (3,5) | 1/9 (11.1) | 7/9 (77.8) | 1/9 (11.1) | Participants felt that this is a good guiding document on what components should be present in a successful TiC but felt that this was more a manual than tool and that completing all items would be onerous. Participants liked the inclusion of community support systems. If this tool was to be modified for the ICU, it should be created with patients and families, and include a checklist that could be completed throughout the patient’s ICU stay. |
| Discharge Planning Questionnaire [30, 31] | ICU nurses | Critically ill elderly patients | 51-item screening questionnaire used by ICU nurses to assist in determining critically ill elderly patient’s perceptions of discharge needs and outcomes | 37 (35,39) | 4 (2,5) | 1/8 (12.5) | 3/8 (37.5) | 4/8 (50) | Participants felt that this TiC tools is good for patients going home from the ICU but is targeted towards elderly patients and should be adapted so it includes a broader patient population. Though some liked that it opens the conversation about ADLs, others felt these questions were not relevant because the care team cannot do anything with the information (e.g., rehab, homecare) and patients and families may not feel heard or that it is a waste of time |
| Nursing Care Management [33] | Nurse practitioners as case managers | NICU patients and families | A pathway to approach the care and discharge planning for babies in the NICU | 42 (39,47) | 5 (5,6) | 2/8 (25) | 6/8 (75) | 0/9 (0) | Participants liked the cartoon because there was comfort in seeing a graphic of the TiC process. Though, many felt the NICU tool was not applicable to critically ill adults, because there are not a standard set of milestones that are applicable to a general ICU setting. Modifications to this tool should include modernizing the cartoon, co-designing a TiC pathway with patients and families which can be individualized and includes goals or milestones. Though caution should be taken as some patients may move back a step. |
| Discharge Planning Train [35] | NICU staff | Patients and families | Method to identify and categorize neonatal medical needs and risk factors at the time of discharge from the NICU | 46 (44,49) | 5 (5,6) | 1/9 (11.1) | 7/9 (77.8) | 1/9 (11.1) | Participants liked that this tool was visual, transparent (i.e., tracking patient condition), and that discharge planning is started ahead of time. Modifications to this tool should include adding extra boxes (e.g., psychosocial, family readiness, mobility), ability for boxes to be changed to be patient specific, and clear guidance on when the boxes should be updated (e.g., after rounds). Though, participants were concerned about family anxiety if a patient is bumped down to red or if a patient doesn’t have all green boxes but is transferred due to an emergency. |
| Back Transport [22] | NICU staff | NICU patients and families | Reviewed literature on safety and cost-effectiveness of back transport for NICU patients and impact on family caregivers. Described a process to ease the transition between the NICU and community hospital for infant, family caregivers and staff | 43 (40,51) | 5 (3,5) | 0/10 (0) | 7/10 (70) | 3/10 (30) | Participants perceived that this summary would be beneficial for a receiving healthcare provider. Though, they felt back transport was onerous to complete, duplicates current processes, and was too clinical (i.e., patient- and family-focused). |
| Discharge from the ICU to Ward Brochure [26] | PICU liaison nurse | PICU patients and families | PICU liaison nurse-driven intervention comprising a written information brochure for parents of pediatric ICU patients | 44 (42,46) | 5 (3,5) | 1/8 (12.5) | 5/8 (62.5) | 2/8 (25) | Stakeholders like that the language is a positive step in the recovery and may address patient and family anxiety. They also like that there is something written to refer to, but felt that this could not stand alone as a TiC tool. Suggested modifications include co-designing with patients and families, improving language, and including graphics (i.e., too many words). |
| Discharge Summary [34] | NICU | Patients and families of the NICU | Compared computer-generated discharge summaries to dictated summaries and evaluated their satisfactory and suitability for parents | 41 (39,42) | 5 (3,5) | 0/9 (0) | 7/9 (77.8) | 2/9 (22.2) | Participants liked that this tool included prognosis and residual disability and could see the benefit for it going from provider to provider, though felt this could not stand alone as a TiC tool. Modifications should include making it more patient- and family-focused, not making it computer-generated (unless you could make a patient/family-facing and provider-facing version), and including something written you could refer back to. |
| Potentially Better Practices (PBP) [24, 25] | NICU staff | Patients and families | Outlines five PBPs that NICU can adapt in a local context to create a successful discharge planning process that spans the NICU stay to the next level of care | 53 (46,58) | 6 (5,6) | 2/10 (20) | 6/10 (60) | 2/10 (20) | Participants liked that the PBP was inclusive of the NICU team and family and that you could track a patient’s progress. Though, they felt that this may not stand alone as it does not consider the receivers of the TiC. Modifications should include adapting it from a NICU to ICU population. |
| Structured Transfer Brochure [29] | Family member of patients in ICU/ICU care team | Adult ICU | A structured conversation with the use of a brochure between the ICU nurse and family caregivers to reduce anxiety and uncertainty regarding ICU transfer | 55 (53,62) | 5 (5,6) | 1/10 (10) | 8/10 (80) | 1/10 (10) | Participants liked the use of images, that the brochure wasn’t too text heavy, and felt like this was a good start for introducing the ICU and what will happen throughout a patient’s stay. Modifications should include adapting it for the site, translating it to languages, and adapting it so it can be individualized to the patient. |
| Information booklet [19] | ICU care team, patients and families | ICU patients and families | An evidence-based information booklet for patients and family caregivers preparing for transfer from the ICU to augment verbal information | 60 (56,64) | 6 (5,6) | 5/10 (50) | 5/10 (50) | 0/10 (0) | Participants liked that this included diary pages to record events to talk about later, felt that it was respectful of the patient and family, and that its simple structure allowed it to be individualized to each patient. Participants disliked that it included one-way communication and appeared to be all text. Modifications should include translating it to different languages and including graphics (not just text). |
| Safety Checklist for Discharge Planning [18] | NICU nurses | NICU patients and families | A safety checklist of discharge topics for parents to be taught by NICU nurses to reduce accidental infant deaths after discharge | 43 (41,46) | 5 (3,6) | 1/9 (11.1) | 4/9 (44.4) | 4/9 (44.4) | Participants liked the concept of teaching caregivers things they can do to help the patient transitions and how to provide ongoing care. Though, participants felt this tool could not be adapted for an ICU population and felt it did not include enough items or context. If this tool is modified, it should be completed in collaboration with patients and families and should include topics that ICU patients are at risk for, including teaching of what to look out for and what to do. |
| Neonatal Discharge Assessment Tool (N-DAT) [36] | Multidisciplinary NICU team | NICU patients and families | Method to identify and categorize neonate medical needs and risk factors at the time of discharge from the NICU | 37 (34,43) | 3 (2,5) | 2/9 (22.2) | 3/9 (33.3) | 4/9 (44.4) | Though participants thought the included domains represented all what is involved in caring for an individual, they felt this 75-item checklist is too long for patients and families and too onerous to complete. |
| Pediatric Acute Burn Discharge Planning Index [32] | Health professionals | Pediatric burn patients and families | Developed a discharge planning index for pediatric acute burn patients based on expert opinions | 45 (43,47) | 6 (5,6) | 2/8 (25) | 5/8 (62.5) | 1/8 (12.5) | Participants liked that this was a check list, included categories with spaces for comments, involved the family, easy to hand off (i.e., between shifts or providers), fit with the workflow, and included respiratory, physiological, and sleep-related items. Modifications include adapting it for and ICU and ensuring that assessments for each item are completed in the same manner. |
| NICU discharge DVD/ Video [27, 28] | NICU patients and families | NICU patients and families | A 35-minute, evidence-based, NICU discharge DVD used to educate patients who do not attend in-person NICU discharge classes | 31 (29,38) | 3 (2,4) | 0/8 (0) | 2/8 (25) | 6/8 (75) | Participants felt that this is too long, not a standalone tool and, because it is a video, not modifiable for different patient conditions or situations (e.g., patients without families). If this was to be modified, participants suggest making it a digital story as an introduction to the ward and that it be codesigned with patients and families. |
| Discharge Planning for AIDS patients [17] | Critical care nurse | AIDS patients | A guide specific to individuals with AIDS to conduct discharge planning from ICU/ hospital to home that assesses patient knowledge and provides patient education | 44 (39,50) | 3 (2,4) | 0/10 (0) | 5/10 (50) | 5/10 (50) | Participants liked that this was comprehensive and could be used as a guide. Participants disliked that this included only “one way” communication, was overwhelming for patients and families to receive, onerous for a member of the care team to deliver, included dated language, and was specific to AIDS. Modifications would need to include a communication component and adapting the guide for every single disease. |

Abbreviations: ADL, Activities of Daily Living; ICU, Intensive Care Unit; NICU, Neonatal Intensive Care Unit; PICU, Pediatric Intensive Care Unit; TiC, Transitions in Care
