# Supplementary material for: Co-development of a transitions in care bundle for patient transitions from the intensive care unit: a mixed-methods analysis of a stakeholder consensus meeting
Source: BMC Health Serv Res. 2022 Jan 2;22:10. doi: 10.1186/s12913-021-07392-2 (PMC8722038; doi:10.1186/s12913-021-07392-2)
Supplement: Supplementary file 2 — Additional file 2. [file 12913_2021_7392_MOESM2_ESM.docx]

**Additional file 2.** Qualitative themes and subthemes from breakout discussions

| **Theme** | **Stakeholder** | | | | | **Example quote** |
| --- | --- | --- | --- | --- | --- | --- |
|  | **Patients** | **Healthcare providers** | **Decision makers** | **Researchers** | **Knowledge-users** |  |
| **Barriers** | | | | | | |
| Health system capacity | ✔ | ✔ | ✔ |  | ✔ | *“The lack of resources, the lack of time, the strain on capacity in terms of beds or staff makes even the best constructed, most well co-designed transition bundle challenging to implement and sustain.”* – Decision Maker |
| Health system culture |  | ✔ | ✔ | ✔ | ✔ | *“If we don't get underneath the root-cause issue of the culture change that needs to happen, there's ... really no point in doing all of this. Because it'll be another tool, another app, another discharge form, right? … But coming head on at the culture is also difficult. So, how do we backdoor a culture change with the use of a bundle?”* – Knowledge-User |
| Patient and family capacity | ✔ | ✔ |  |  |  | *“I mean you have a patient who's not ready. Many of them have really invested families. Some of them don't. And so if your patient's not ready, but your family is, you can educate them, mobilize them to kind of take that role until the patient's ready.”* - Nurse |
| Varying levels of family presence | ✔ | ✔ | ✔ |  |  | *“[S]omeone who is homeless and is being discharged to… a very different circumstance than someone who is… a 21 year old with their parents at the bedside every day versus an elderly person who's going home with home care support.”* - Patient |
| **Facilitators** | | | | | | |
| Adaptability | ✔ | ✔ | ✔ | ✔ | ✔ | *“It's very detailed, which for me it's really good and it seems that you could quite quickly edit it to on a case by case basis. It provides a lot of detail, a lot of fields. So, it seems to be that it can be easily edited to be customizable [to the] patient.”* – Patient |
| Champions and provider buy-in |  | ✔ | ✔ |  | ✔ | *“[W]ith providers, I think one of the biggest thing that's a big facilitator is knowing why we're doing this and why it's important because you're not going to get buy in from any provider unless you can really sell them on this is why we need to do this and this is why we're working on it.”* – Decision Maker and Nurse |
| Collaboration and co-design | ✔ | ✔ | ✔ |  | ✔ | *“I think making sure that [there is] that multidisciplinary component to this. … there's certain things that as a physician or as a nurse, the ICU team are really important for us to be thinking about. But then there's also things that really matter to the patient and the family, or from a social work perspective, or a transition services perspective. So [ensuring] that, whatever those important pieces are and are all involved in that transition.”* - Patient |
| Comprehensiveness and consistency | ✔ | ✔ | ✔ | ✔ | ✔ | *“As long as the information remains consistent to what is being verbally said and what's on the paper. If you have somebody that's heavily medicated, they're still able to read but they might not get what is being asked of them. … The written document as well to refer back when they're not, say, in a less compromised state of mind.*” - Patient |
| Education for discharge expectations | ✔ | ✔ | ✔ | ✔ | ✔ | *“Another thought we heard frequently is patients and family members had no idea what criteria had to be met or what wellness of the patient needed to be met in order for them to even get out of ICU. That's part of the expectations. We want you to progress to whatever that looks like and then you're at [that] point ... it sets the expectation.”* - Patient |
| Integration into existing care pathways | ✔ | ✔ |  | ✔ | ✔ | *“[U]understanding how the system works and building your tool to fit with it as easily and as flexibly as much as possible. [B]uilding the tool to fit with … the infrastructure we have as opposed to building a tool and then trying to fit it into the infrastructure.”* – Researcher |
| Trust and transparency | ✔ | ✔ | ✔ |  | ✔ | *“…the discussions really highlight the benefits and the importance of co-designing any tool kit that I think always both sides need to be together … the transparency that transparency is a big issue and that I think both sides agree. … I think it really speaks to transparency that both providers want to be transparent and both patients and families want transparency. … If you keep that as your main focus and modify a lot of those things, I think we're going to come up with a great toolkit.”* – Decision Maker |
| **Implementation Considerations** | | | | | | |
| Continuity | ✔ | ✔ |  |  |  | *“The other piece we talked about was the importance of some continuity. Meaning that … once that transition takes place … this patient and this family go from the ICU settings to a hospital ward or to home, having some piece that can travel with them so that the pieces that are started to facilitate a smooth transition aren't lost.”* - Patient |
| Continuous evaluation and improvement | ✔ | ✔ |  | ✔ | ✔ | *“[A]dditional material to present … if someone was to try to implement this and study it would be … what measures do you need? What should you be measuring to ensure that [you] can see how your implementation works compared to ours for example, and then information on both the development and evaluation? That being explicitly like what worked well, what didn't work well, learnings from that. And then … maybe it's like evaluation of the bundle of clinical outcomes … [T]hese are the outcomes of people who had the full bundle. These are the outcomes that people who had only some of the bundle and the people who didn't get it at all.”*- Facilitator |
| Delivery | ✔ | ✔ |  | ✔ | ✔ | *“I think the value of a tool like this comes a little bit in the context in which it's provided, which are the people you've already mentioned. There's something I've seen some other teams do as they started with the concept … but then adapted it into a video or … another format that's maybe a bit more engaging, or offers that chance for that information to be transferred to have that discussion if there is still some questions. And then like keep their brochure for afterwards to follow up.”* - Patient |
